# Exploring mental health practice among Traditional health practitioners: a qualitative study in rural Kenya

**DOI:** 10.1186/s12906-018-2393-4

**Published:** 2018-12-14

**Authors:** Christine W. Musyimi, Victoria N. Mutiso, Lianne Loeffen, Anja Krumeich, David M. Ndetei

**Affiliations:** 1 Africa Mental Health Foundation and Department of Psychiatry, University of Nairobi, Mawensi Road, Off Elgon road, Mawensi Garden, P.O. BOX 48423-00100, Nairobi, Kenya; 2 Vrije Universiteit, 1081 HV Amsterdam, Netherlands; 3 Maastricht University, P.O. Box 616, 6200 MD Maastricht, Netherlands; 4 University of Nairobi, P. O. Box 30197 00100, Nairobi, Kenya

**Keywords:** Traditional health practitioners, Traditional medicine, Mental health, Rural, Kenya

## Abstract

**Background:**

Involvement of traditional health practitioners (THPs) in the form of collaboration with the formal health care system is suggested to improve the pathways to mental health care in Kenya, yet understanding of the current traditional practice and THPs’ perspectives is lacking. The aim of this study was to explore the views of THPs with respect to their mental health practice.

**Methods:**

This study qualitatively explored the views of THPs, using four focus group discussions (FDGs) each consisting of 8–10 traditional and faith healers, resulting in a total of 36 participants. Thematic content analysis using a grounded theory approach was performed using QSR NVivo 10. Emerging topics were identified and examined by re-reading the transcripts several times and constantly re-sorting the material.

**Results:**

Four themes that reflect THPs’ mental health practice perspectives emerged as follows: 1) Categorization of mental illness; 2) Diagnostics in traditional mental health practice; 3) Treatments and challenges in current traditional mental health practice; and 4) Solutions to improve traditional mental health practice.

**Conclusions:**

These themes provide insight into the perspectives of Kenyan traditional and faith healers on their mental health practice, in an attempt to offer a meaningful contribution to the debate on collaboration between informal and formal health care providers in improving mental health services in Kenya. Furthermore, the presented challenges and solutions can inform policy makers in their task to improve and scale up mental health services in resource-poor areas in Kenya. Addressing these issues would be a first step towards understanding the solid foundation of traditional medicine that is necessary before collaboration can be successfully attempted. Further research is also recommended to assess patients’ needs and explore potential forms of collaboration, in order to achieve sustainable improvement in the mental health care pathways for patients.

**Electronic supplementary material:**

The online version of this article (10.1186/s12906-018-2393-4) contains supplementary material, which is available to authorized users.

## Background

Mental disorders currently account for one-third of the global disability and are among the leading causes of disease burden [1]. In Low and Middle Income Countries (LMICs), mental disorders account for 25.3% to 33.5% of all years lived with disability [2]. A recent meta-analysis also demonstrated that they are among the major causes of death globally, suggesting a need to prevent this occurrence in vulnerable populations [3]. Despite these statistics, mental health still receives insufficient attention in LMICs due to competing health priorities and limited resources [4–6].

Kenya is one of the developing countries facing the challenge of high mental health burden [7]. Almost half of the patients visiting a health facility suffer from a mental disorder with estimates of about 10% in the general population [7–9], compared to about 40% or less in other LMICs [10, 11]. Notwithstanding, mental health services in Kenya are still relatively underdeveloped due to lack of resources; brain drain (migration of trained health workers to other countries in search of better pay, living conditions, technology and stable political conditions); and underdeveloped policy-frameworks; which result into challenges in delivery of population-based mental health services [6, 12, 13]. On an estimated population of 40 million people, there are only 78 active clinical psychiatrists, amounting to a psychiatrist-to-population ratio of 1:512.820 [14]. The treatment gap is amplified even more as most of the mental health professionals are located in urban areas and private practice, which are not accessible to the majority of the population (more than 70%) who live in rural areas [14, 15] and rely mainly on traditional health practitioners (THPs). Community members and local health providers such as THPs and community health workers in these settings may not have the terms for specific types of mental illnesses but are able to describe its symptomatology. For instance, psychosis would be termed as “madness”, depression as “thinking too much or stressed” and epilepsy as unnecessary falls [16–18]. The community members mainly attribute mental illness to sorcery or displeased ancestral spirits [18, 19] and therefore, direct patients to receive mental health care from THPs [17]. It is thus important to note that unless alternative solutions are sought to reduce this gap or avail the necessary resources to further understand the traditional African models of detecting mental illness, the burden of mental illness is expected to grow, which indicates the need for change and improvement of mental health care pathways in Kenya [4, 12].

Various studies have indicated that a potential solution should include the involvement of the informal health care system, which works actively alongside the formal biomedical health care system in Kenya [6]. It consists of indigenous traditional and faith healers, who are referred to as THPs, exerting an ancient and culture-bound method of healing that has evolved during centuries of practice [20]. Traditional healers consist mainly of herbalists and witch doctors, and commonly use herbal medication or perform rituals and counseling to treat their patients [18]. On the one hand, traditional healers do not receive any formal education but they inherit their practice through possession by ancestral spirits or gain experience through on-the-job training [21]. Faith healers on the other hand use prayers, bible interpretation and counseling as modes of treatment [21]. Their education varies from participating in seminars, workshops and sometimes higher levels of education ranging from diplomas and degrees to doctorate. However, those practicing in rural areas mostly lack basic education. Healers in Kenya are generally recognized by the government through the Traditional Health Practitioners’ bill [22] but do not formally refer patients to hospitals. This bill is considered as one of the ways in which ‘fake’ and ‘genuine’ healers are differentiated. Research shows common perceptions of ‘fake’ healers as being ignorant and that they tend to administer the wrong medication without following the correct treatment regime [23] or use false doctrines to financially exploit their congregants.

Almost 50% of the population in Africa consult traditional and religious healers for treatment of mental illness before accessing formal biomedical health care [24]. More specifically, a study in Kenya found that the majority of patients with mental illness in Nairobi consulted both Western medical practitioners and THPs [9]. The popularity of these practitioners can be attributed to their affordability, flexibility and accessibility - as the ratio of 1:250 is much better compared to one medical doctor for every 70.000 people [20, 25]. Considering the current treatment gap in mental health care and the high numbers of patients that choose to visit THPs, it has been suggested that involvement of THPs is vital in the improvement of mental health care in Kenya [6]. Other researchers have also stated that the inclusion of THPs would improve the accessibility of care and meet the need and wish for cultural sensitivity in scaling up mental health care [13, 26].

Collaboration and referral between the formal and informal health systems has been the focus of recent studies that aim to explore how the two could complement each other [6, 19, 27]. Despite the persistent attention for THPs throughout the last 50 years that has acknowledged their importance but also critically pointed out the challenges that separate them from the formal health care providers, what is often missing is an understanding of the perspectives of THPs [14]. If the mental health care system in Kenya is to be successfully improved by involving THPs and setting up collaboration with the modern biomedical practices, it is vital to understand the current traditional practice and the perspectives of THPs. There is lack of recent qualitative studies that investigate traditional mental health practice in Kenya from their point of view, which has left a knowledge gap that the current study aims to fill.

This study serves as a baseline-study aiming to qualitatively explore the views of traditional and faith healers in rural Kenya, where mental health workforce is especially lacking [28]. It sets forth their perspectives on the current mental health practice in an attempt to obtain a better understanding of the current situation of traditional mental health practice, in order to contribute to the debate of potential collaboration between the formal and informal health care providers.

## Methods

The aim of this study was to explore the perspectives of traditional and faith healers on their mental health practice in rural Kenya. It was conducted in May 2014 within four randomly selected regions in Makueni County, one of the 47 counties in Kenya. The county consists of arid and semi-arid areas and is situated in Eastern Kenya. At inception of the project, a total of eight Focus Group Discussions (FGDs) consisting of traditional healers (*n* = 2), faith healers (*n* = 2), clinicians (*n* = 2), a group of traditional healers and clinicians (*n* = 1) and finally a combined group of faith healers and clinicians (*n* = 1) were conducted[19]. As the current study aimed to explore only the perspectives of THPs, four (strictly for traditional healers (*n* = 2) and faith healers (*n* = 2)) of the eight FGDs were selected for data analysis, resulting in a total of 36 participants. There were no new themes that emerged from the analysis during the second FGD of traditional healers and faith healers that warranted additional groups. However, it is important to note that a total of four FGDs (traditional healers and faith healers) for THPs were included in the analysis. This complies with a recent recommendation by Guest and colleagues [29] to conduct between three to six FGDs in order to ensure 90% discovery of themes. The rest of the FGDs which involved formal health workers and two other independent discussions with traditional healers and faith healers with clinicians in each of the groups discussed mostly on perspectives particular to formal health workers and collaborative strategies identified in the first four FGDs which have been outlined in an earlier published article [19], hence their exclusion in the current analysis.

Each FGD consisted of 8–10 participants to ensure variation of opinions, and lasted between forty minutes to one hour. The healers that were included in the FGDs were selected through simple random sampling, and all groups had a male to female ratio of 1:1. The sampling was done by shuffling numbered papers from a list of 25 males and 25 females (a total of 50 healers) that were willing and available to participate in the discussions on the suggested days of data collection as provided by the region representative [19]. These healers were initially contacted by telephone from a list of registered healers as per the traditional health practitioner’s bill and faith healers affiliated to a place of worship neighboring the study site. The region representative then forwarded the names of the first available 25 males and 25 females to the project officer who randomly selected between 8 and 10 participants for participation in each of the FGDs. The regions from which the data were collected comprised of about 100 registered traditional healers and more than 100 faith healers affiliated to a place of worship.

As per the methods of the initial study, the questions and guidelines for the FGDs (attached as Additional file 1) were constructed by a group of community mental health researchers and lay workers, covering topics that assessed views on: the definition and causes of mental illness; encounter with patients with mental illness; interaction and enhancement of collaboration with other practitioners; and barriers and solutions in this interaction as well as in the care for their patients [19]. The discussions were open to any additional topics that were brought up by the participants and additional questions were added if clarification was necessary. The FGDs were audio taped and scribes were present during all discussions to actively take notes, capture information that could not be translated into text from the audiotapes such as any form of body language, in order to observe any unusual behavior during discussions.

The FGDs were all conducted indoors for privacy and anonymity reasons and were led by a researcher who was fluent in the local language ‘Kikamba’ and experienced in community mental health and conducting qualitative interviews. Participants were not paid to participate in the discussions, however, transport reimbursements were provided at the end of discussions. Information in the audiotapes was transcribed and translated into English for analysis purposes.

### Data analysis

The English transcripts of the four selected FGDs were analyzed using the grounded theory approach, and open coding used to identify themes [30]. Thematic content analysis was done with use of QSR NVivo 10, where traditional and faith healers were categorized to enable analysis of codes by participant type. Emerging topics were identified and examined by re-reading the transcripts several times and constantly re-sorting the material. After initial open coding, the researcher engaged in focused coding, selecting what seemed to be the most useful initial codes [30]. This led to the final core themes that were meaningful in an attempt to obtain a better understanding of the current situation of traditional mental health practice, after which the researchers categorized the data within the themes.

## Results

Upon analyzing the data, traditional and faith healers revealed similar practices as mentioned within the four emerging core themes that reflect the mental health practice of THPs in rural Kenya: 1) Categorization of mental illness; 2) Diagnostics in traditional mental health practice; 3) Treatments and challenges in traditional mental health practice; and 4) Solutions to improve traditional mental health practice.

**Fig. 1:**
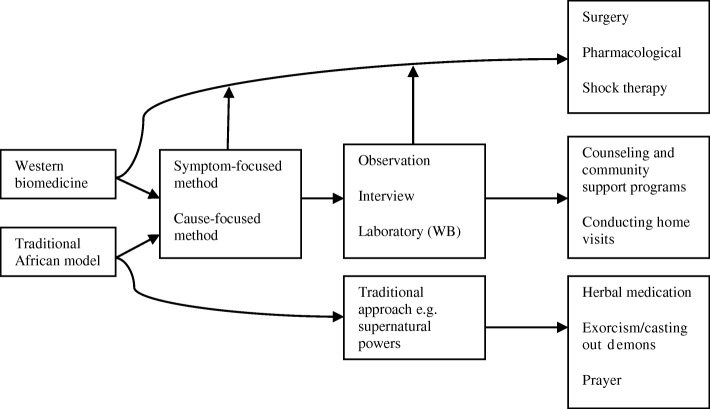
Relationship between Western biomedicine and Traditional African Model. Both Western Biomedicine (WB) and Traditional African Model (TAM) use similar methods to identify the cause of mental illness and to provide treatments for mental illness. The specific treatment modalities include herbal medication, casting out demons and prayer for TAM; and surgery, pharmacological and shock therapy for WB. In some instances a verbal (undocumented) referral for laboratory testing is made if during TAM the practitioner realizes this need. Traditional approaches such as consulting supernatural powers are also employed in TAM particularly if the cause is attributed to witchcraft

The first theme provides insight into how THPs view mental illnesses, by showing how they categorize them. The second and third themes display their practices in the diagnostic and treatment stage which may have an effect on how mental illness is categorized and communicated to the patients and their families. These themes serve well to understand THP views and practice of mental health, and reflect on the challenges they currently experience in trying to manage their patients suffering from mental illness. In light of those challenges, the final theme covers the potential solutions that are put forward by the practitioners (Fig. 1).

### Categorization of mental illness

The data revealed that THPs categorized mental illnesses using either *symptom-focused method* or *cause-focused method,* or sometimes both methods in the same patient, which reveals a glimpse of the way they understood the phenomenon. Firstly, in the *symptom-focused method of categorization*, healers distinguished mental disorders based primarily on the presented symptoms by the patient. Herein, the healers used their own knowledge base that contained information on symptom-patterns and the mental illness label that fits those symptoms, as described below:

“If it is madness, the person runs aimlessly. If it is depression, the individual is disturbed in their mind and does things they are not supposed to do.” (Faith Healer, FGD 1)

Secondly, the *cause-focused method of categorization* represents the alternative way THPs categorized mental illnesses, wherein they focused on the cause of the symptoms or illness presented by the patient. This method reflects the many different ways that THPs attributed mental illness, which were seen to include both ‘traditional beliefs’ such as witchcraft and ‘biomedical beliefs’ that resembled Western biomedical perspectives for instance malaria and impacting life-events. THPs explained that mental illness can have different causes in different people and emphasized on the importance of distinguishing them as follows;

“First type is mental illness as a result of normal sickness which causes brain damage. The other is caused when a person falls or is beaten and the third is linked to malaria.” (Traditional Healer, FGD 2)

“Madness coming as a result of smoking bhang is different from madness coming as a result of being bewitched. Another cause of mental disorder comes in the form of four internal diseases, namely yeast infection, candidiasis and cervical cancer.” (Traditional Healer, FGD 1)

### Diagnostics in traditional mental health practice

The diagnostic methods described by the THPs could be subdivided into four categories: observation, interview, laboratory approach and traditional methods. The use of these methods varied widely among practitioners and some also combined several diagnostic methods. It is possible to draw parallels between the first three categories and the Western biomedical diagnostic system, which implies the presence of both modern and traditional theoretical discourses in the current mental health practice of THPs.

The first diagnostic method is *observation,* through which THPs obtained information about the presented symptoms. Reflecting the *symptom-focused method* of categorizing mental illnesses, the practitioners used their knowledge to set a diagnosis by linking the observed symptoms to the appropriate mental illness label.

“We identify with their physical appearance, if a person was strong and now they have become weak, we definitely know there is a problem.” (Faith Healer, FGD 2)

“Depression is expressed by the individual being silent and looks worried. When they come to us we look at their facial expressions, do they look stressed? Do they appear worried? Do they have a behavior of remaining silent?” (Traditional Healer, FGD 1)

The second diagnostic method consisted of assessing patients by means of an *interview,* as stated by two healers below. The THPs obtained information about the problem, symptoms and probable causes to consequently make a diagnosis. This method thus reflects both the *symptom-focused method and cause-focused method* of categorization, as it distinguishes between mental illnesses and subsequently sets a diagnosis, all based on information about the symptoms and cause at hand:

“You can do a diagnosis of a patient depending on how they have explained their illness or how the people accompanying them have explained to us. You can also know through their life history and the happenings in their past life.” (Traditional Healer, FGD 2)

“We identify the type of illness from our discussion with them and from our interaction with them.” (Faith Healer, FGD 2)

The third category is the *laboratory approach*, whereby THPs referred patients to hospitals or other facilities for laboratory tests, in order to determine the underlying biomedical issue. This was either used as the primary diagnostic or supplementing other methods and it reflected the *cause-focused method* of categorizing mental illness with an emphasis on detecting physical clues that may explain the cause of the mental illness:

“If the patient comes and is silent or is unable to express themselves or explain their illness, we normally send them to the laboratory for tests in the hospitals or to other people who carry out the tests. After the results, we would know what herbal medicine to give.” (Traditional Healer, FGD 2)

The final category contained methods that stemmed from a purely traditional discourse, where a variety of spiritual and supernatural methods were used to determine the patient’s problem. The exact method, however, depended entirely on the abilities of the specific healer, as stated below. This diagnostic category did not fit into the symptom- or cause-focused method of categorization as straightforwardly as other diagnostics. Using traditional diagnostics, the practitioners seemed to have a way of categorizing mental illnesses that is not easily described;

“I can read God’s word and pray to God for Him to reveal to me the problem facing the individual.” (Faith Healer, FGD 2)

“The gods in me show me what people are suffering from even before they arrive. Whether it is sickness, family problems, marital problems or witchcraft, whichever the problem I am shown by the gods in me.” (Traditional Healer, FGD 1)

This broad overview revealed the variety of diagnostic methods that were used by THPs in their mental health practice. We found some individual differences and inconsistencies, and it was therefore questionable to speak of an actual diagnostic system within traditional mental health practice. These inconsistencies seemed to stem from the practitioner’s individual properties, such as experience, abilities, knowledge, intuition, and possibly other factors. All these properties influenced and guided the process of managing patients with mental illness and the choice of diagnostic method.

### Treatments and challenges in traditional mental health practice

The participating THPs described that after establishing a diagnosis, they would carry out the treatment that they deemed appropriate. Even though THPs generally had their own specialization, for instance, herbalists focused on the use of herbs as a treatment modality and witch doctors used their spiritual skills to expel mental illness, they combined different types of methods as well. Counseling was used to treat depression, whereby listening was considered crucial for relieving stress and thus recovery. Among faith healers, the commonly used methods varied from counseling and prayer, casting out demons and conducting home visits to offer additional help. The following statements illustrate how THPs used different forms of treatments for patients suffering from mental illness.

“Modes of treatment will differ depending on the individual and they vary from use of herbs, spiritually removing the mental illness, counseling, rehabilitation and medical attention.” (Traditional Healer, FGD 1)

“…For the demon possessed, we cast out the demons but for those with stress we do counseling.” (Faith Healer, FGD 2)

“…we pray for them, we encourage them, we share the word with them and show them that there is hope in Jesus. You go further and offer them financial support, clothe them and feed them.” (Faith Healer, FGD 2)

In every new situation and with every new patient, the THP decided from the pool of treatment options which one was most appropriate, depending on the following factors: the type of mental illness, the cause of the illness, the healer’s knowledge of mental illnesses and corresponding treatments, the type of healer or his/her specialization, and the healer’s former experience. Individual context and personality thus played a vital role in this decision process.

Traditional health practitioners also mentioned several challenges that they had experienced in their practice, which included inadequate financial resources to run their business, lack of knowledge and skills to treat mental illness, patient-related challenges and untrained/poorly trained practitioners.

Firstly, many of the healers clearly stated that their mental health practice was hindered by lack of financial resources. Some healers that relied on tests or scans at the hospital to assess a patient’s illness were unable to buy the necessary machines for performing the tests, and also faced a challenge in maintaining a decent office for receiving patients. Healers that used herbal medicines as a form of treatment also struggled to purchase herbs due to a lack of operating capital. Moreover, faith healers conducting home visits did not have finances to facilitate their transport.

“We lack capital to buy medicine. We also face the challenge of machines for carrying out tests on patients. We have offices which are not so conducive and we lack money to make decent offices and this may be a problem with the health inspectors who might close the offices.” (Traditional Healer, FGD 1)

“We need some support allowance to facilitate our mobility in the community as we conduct home visits and refer the sick to hospitals.” (Faith Healer, FGD 2)

Secondly, lack of knowledge or skills often occurred when practitioners were presented with new situations, and it implied that they were only able to handle some conditions based on their prior experience:

“… I only treat sicknesses that I know I can treat. Those that I don’t know what they are I don’t treat.” (Traditional Healer, FGD 2)

“…We do not have the knowledge especially on a new occurrence or situation that we have never encountered or handled before.” (Faith Healer, FGD 1)

Thirdly, THPs experienced issues related to patients’ attitudes which resulted in patients mistrusting the healer and their practice. Patients were also reluctant to visit the hospital even after being referred by the healers as their beliefs conflicted with those of the healer - particularly around illness causation. The following statements illustrate these attitude issues;

“If a patient believes their sickness is due to demons, they refuse to go to hospital even when we refer them ...” (Faith Healer, FGD 1)

“We get educated people who come for our services and they demand to know our level of expertise by asking us to show them our certificates of practice before they can trust us and pay for the services.” (Traditional Healer, FGD 2)

In addition, practitioners were not able to adequately diagnose or treat patients who were unable to pay for tests at a hospital. As a result, healers took up the responsibility of paying for the costs, which in turn contributed to the practitioner’s financial burden:

“Some patients don’t have money to go to the hospital and we have to incur the expenses when we refer them.” (Faith Healer, FGD 1)

Practitioners explained that some patients’ relatives counteracted proper treatment by being uncooperative hence becoming a hindrance to their mental health practice:

“If the mental illness is as a result of witchcraft and you tell their people, some family members refuse to allow us to continue with the treatment for reasons unknown to us …” (Traditional Healer, FGD 1)

Lastly, a major issue for THPs was the existence of ‘fake’ healers, whose malpractice counteracted genuine practitioners. Registered/genuine herbalists lost clientele as ‘fake’ healers sold their medicine at cheaper rates and advertised their practice by playing music and dancing in the open air. ‘Fake’ herbalists were said to lack proper training for the herbal medicines and often made improper dosages for herbs, which resulted in negative outcomes and patients mistrusting all herbalists. Cases of malpractice resulted in a bad reputation, in turn causing mistrust from patients and their families. The following statements illustrate the issues of malpractice:

“We also have herbalists who move around selling medicine and giving the wrong dosages and when the medication is not effective, people view and generalize that all herbalists are ‘fake’ and their medicine is not effective and we lose out on potential clients.” (Traditional Healer, FGD 1)

The challenges of ‘fake’ healers, malpractice, and bad reputation were mostly mentioned by traditional healers.

### Solutions to improve traditional mental health practice

In light of the challenges experienced in traditional mental health practice, the THPs suggested several measures to resolve the issues in their practices. These solutions included; existence of rules and regulation, provision of training, enhancing awareness, collaboration and embracing religion.

Firstly, according to traditional healers, rules and regulations were needed to address the problem of malpractice, which in turn would aid in alleviating the patient’s mistrust. It was suggested that all practicing healers needed letters of operation from the relevant authorities, documents needed to be screened, and medicines exposed to regular tests. The provision of certificates and license were also important to help enhance the patient’s trust in the practitioner as stated below;

“Fake herbalists don’t have letters of operation from the authorities. They should be banned from operating until they go for training. Their medicine should be taken for testing because when they administer poisonous medicine, they give us a bad name and we lose business. Their documents should be screened.” (Traditional Healer, FGD 1)

“…To build trust about your treatment and the herbal medicine that you use, you can give your certificates and licenses to those doubting your practice and operations.” (Traditional Healer, FGD 2)

Secondly, THPs stated the importance of being trained on good mental health practice, and suggested that it should be offered to prevent patient mistreatment. While training would expand the practitioner’s knowledge and skills, their ability to manage and treat different types of mental illnesses could increase. This addressed the issue of THPs who mentioned challenges related to lack knowledge and skills in handling some mental illnesses.

“We should have unity amongst us, teachers, herbalists, doctors, and witch doctors in order to work harmoniously. Training should be conducted for all of us to avoid friction and mistreatment of patients.” (Traditional Healer, FGD 2)

“We should be taught about offering first aid to patients before we refer them.” (Faith Healer, FGD 2)

Thirdly, practitioners suggested that more awareness among healers on each other’s practices and modes of treatment could promote knowledge exchange so that if certain healers are not able to handle certain illnesses, they could seek assistance from a colleague with the appropriate set of skills:

“We should hold training together to create awareness and be educated on each other’s modes of treatment and practices. There is something they know that we don’t know and there is something we know that they don’t know. So we need to exchange ideas and know how to collaborate and work together for the sake of the patients.” (Traditional Healer, FGD 1)

Fourthly, practitioners mentioned the potential for collaborating with biomedical health providers, other THPs and the county government to improve their knowledge through training. It was proposed that the County government or local administration could contribute by arranging training to educate healers from sub-counties, as THPs did not often have the means to travel far or pay for extensive training:

“… or you can find other channels like from the county? Or have them make provisions like them coming to train in the counties or they can come to a place like here, train for three days in a week or they just come and fully fund it and train you for three full days especially with all these groups in the sub counties. Isn’t that a good idea?” (Traditional Healer, FGD 2)

Finally, nearly all faith healers stated prayer as the main solution for their problems while applying various principles. Prayer was not stated as a main solution among traditional healers.

“We have to get a spiritual approach to solving the issue, we have to seek God and be able to apply His principles to solve the issue.” (Faith Healer, FGD 1)

## Discussion

This study aimed at providing insight into the perspectives of Kenyan traditional and faith healers on their mental health practice, in an attempt to offer a meaningful contribution to the debate on collaboration between THPs’ mental health practice and the formal health care system in improving mental health services in Kenya. Four themes describing the THPs’ understanding of mental illness, their practice, challenges they experience and potentially fitting solutions emerged.

Practitioners were found to be using both *symptom-focused* and *cause-focused methods* to categorize mental illnesses. These methods represented ways in which they used their knowledge base of mental health, thus showing their theoretical understanding of these issues and expressing their views on mental illness. There are similarities between the described ways of categorizing mental illness and the explanatory model approach of medical anthropologist Arthur Kleinman, which states that every practitioner, from whichever type of health care system, forms an explanatory model for every new patient and every new situation, which consists of ideas and explanations about illness characteristics and treatments [31]. The patient voices the explanatory model while the practitioner elicits this model based on a series of patient-centered questions in order for the practitioner to identify the most appropriate treatment.

Within these methods of categorization, the THPs’ explanations of mental illness were found to contain elements from both traditional and biomedical discourse. This finding contrasts with many studies in the last century that have attempted to describe the traditional African views on mental illness, with early studies claiming that all illnesses are attributed to witchcraft in the end [32]. The focus has often been to relate African indigenous disease theory to supernatural aetiological agents, which has caused many to accept that there is a fundamental dichotomy between Western biomedicine and traditional systems [25, 32]. This study contradicts a strict dichotomy, by showing that the current belief-models in traditional mental health include a combination of traditional and Western discourse. Recent studies have identified this combination [33–36], resulting in many agencies taking a step to improve collaboration between formal and informal practitioners [37].

Common diagnostic methods reported by THPs included *observation, interview, laboratory approach* and *traditional methods*. These processes resemble diagnostics that were described to be ‘traditionally African’ about three decades ago, when the main tools were categorized as follows: detailed history taking, physical examination of the client, and divination [25]*.* The physical examination is slightly modernized as it now consists mostly of laboratory testing. Apart from being traditionally African, however, the first three categories described in this study also clearly resemble methods that are used in the Western biomedical diagnostic system. Indeed, these resemblances support the earlier statements of combined theoretical discourses in traditional mental health practice, while again proving the unclear division between traditional and modern practices.

The treatment phase in traditional mental health practice is decided based on the illness and its established cause. Both these variables are established in the diagnostic phase and depend on variables such as the healer’s knowledge and beliefs of mental illnesses and their causes. The choice of appropriate treatment is also influenced by the specialization of the involved healer, while individual experience and personality may explain the final variation between healers. These treatment modalities are similar to those practiced by THPs in urban settings in Kenya and in other LMICs [18, 38, 39], demonstrating a form of informal uniformity in their practice but existence of modern aspects of diagnosis and treatment. This indicates that the dichotomy between traditional medicine and Western biomedicine may not be so distinct and forms a basis on which collaborative efforts between THPs and formal health workers can be established in the attempt to improve and expand mental health services in Kenya and provide mental health services in low-resource settings. While the lack of standardization is often portrayed as a problematic characteristic of traditional medicine that stands in the way of collaboration [40, 41], the perspectives of THPs demonstrated their willingness towards forms of standardization as a solution to their challenges. A recent study has demonstrated this collaboration by engaging THPs to provide evidence-based treatment [42]. Furthermore, they can participate in conventional mental health service provision or their services blended into the conventional practices to form a new hybrid system that prevents patients from choosing one service over another [43].

The described challenges and potential solutions provide insights into the perspectives of the practitioners, illustrating what they find problematic and which measures they feel would contribute to improvement of their practice. Tackling these issues could be a first step towards scaling up Kenyan mental health services, as it would improve traditional practices and create the solid foundation that is needed before expanding services or collaborating with the formal health care system. Additionally, considering the perspectives of THPs in the process will help create a bottom-up approach to achieve this foundation.

Contrary to the present research, other studies have focused on the challenges of traditional mental health practice from an outsider’s perspective [20, 25]. These approaches differ significantly, as the outsider’s perspective often tends to judge traditional mental health practice by comparing it to biomedical standards. Findings from this study explore the perspectives of those implementing the traditional practice while delving into their challenges and possible solutions for future collaboration.

## Limitations

The use of FGDs to explore the perspectives of THPs potentially elicits socially desirable statements of the participants. Future studies should incorporate other methods of data collection such as key informant interviews to reveal any confidential information that may not be discussed within FGDs. Additionally, as the study was conducted in Makueni County in rural Kenya, generalization to other non-similar settings should be done with caution.

## Conclusion

The presented challenges, solutions and the theoretically grounded model (Fig. 1) can inform policy-makers in their task to improve and scale up mental health services in resource-constrained areas in Kenya by incorporating both Western and traditional African models of understanding, diagnosing and treating mental illness. Addressing these issues would be a first step towards understanding the solid foundation of traditional medicine that is necessary before collaboration can be successfully attempted. Engel and other scholars [44], as well as Kleinman and Cohen [45], argue that THPs may rather possess the much-needed sensitivity to cultural variation that is shown to be vital in mental health, and the availability of diverse forms of treatment may actually increase the chances that a patient finds one that will suit their needs [46]. Additionally, further research is recommended to assess patient needs and explore potential forms of collaboration, in order to achieve sustainable improvement in the mental health care pathways for patients throughout Kenya.

## Additional file

Additional file 1: Focus group discussion guide. This guide covers topics related to the definition and causes of mental illness; encounter with patients with mental illness; interaction and enhancement of collaboration with other practitioners; and barriers and solutions in this interaction as well as in the care for their patients. (DOC 30 kb)

## Abbreviations

FGDs: Focus Group Discussions
GCC: Grand Challenges Canada
LMICs: Low and Middle Income Countries
THPs: traditional health practitioners
