# Supplementary material for: Exploring mental health practice among Traditional health practitioners: a qualitative study in rural Kenya
Source: BMC Complement Altern Med. 2018 Dec 14;18:334. doi: 10.1186/s12906-018-2393-4 (PMC6295025; doi:10.1186/s12906-018-2393-4)
Supplement: Supplementary file 1 — Focus group discussion guide. This guide covers topics related to the definition and causes of mental illness; encounter with patients with mental illness; interaction and enhancement of collaboration with other practitioners; and barriers and solutions in this interaction as well as in the care for their patients. (DOC 30 kb) [file 12906_2018_2393_MOESM1_ESM.doc]

**FOCUS GROUP DISCUSSION GUIDE**

1. What kind of patients do you see in your clinic? Probe for mental illness.
2. What is mental illness?
3. What do you think is the cause of mental illness?
4. What are the different types of mental illness?
5. How would you describe your experience in interacting with people with mental illness in your community? in the course of your work?
6. What do you understand by the term depression? And how is depression different from other types of mental disorders? Do you come across people with depression or low mood in the course of your work?
7. Who should be blamed for a person who has (i) Mental illness (ii) Depression?
   1. How do you identify the problems they have?
   2. What modes of treatment do you use for these patients? On a scale of 1-10, how successful do you think your modes of treatment are?
8. What challenges have you faced when treating your patients? How did you cope with these challenges?
9. Where do you refer patients with mental illness?

- What challenges do you face while referring these patients?
- What solutions do you currently have in mind to mitigate these challenges?
- [**If one does not refer]**, what would be your take in referring patients to a Health Care worker or collaborating with them?

1. What problems do you foresee in collaborating with health care workers? What would be the cause of these problems? How would you like them to be resolved?
